# Structural-color-enabled multispectral heterostructure for infrared and laser camouflage

**DOI:** 10.1515/nanoph-2025-0303

**Published:** 2025-09-04

**Authors:** Wenhao Wang, Long Wang, Tonghao Liu, Yina Cui, Liuying Wang, Gu Liu, Yangming Pang, Xu Wu, Xinyu Zhu, Xiaohui Chi, Haoke Yang, Xiaohu Wu

**Affiliations:** Zhijian Laboratory, 562552 Rocket Force University of Engineering, Xi’an, 710025, China; Shaanxi Key Laboratory of Artificially-Structured Functional Materials and Devices, Air Force Engineering University, Xi’an, 710051, China; Thermal Science Research Center, Shandong Institute of Advanced Technology, Jinan, 250100, China

**Keywords:** multispectral-compatible camouflage, metamaterial, structural color, thermal camouflage

## Abstract

The multimodal detection system has gradually been perfected, essentially covering the entire optical spectrum, posing a significant threat to the survival of objects. To counter this escalating detection threat, the demand for multispectral-compatible camouflage (MCC) is increasingly urgent. However, there are inherent conflicts in the principles of camouflage for visible light, infrared (IR), and lasers, necessitating spectrally selective design to reconcile these conflicting requirements. Here, we propose a multilayer film structure with heterostructure coupling, utilizing resonant cavities, destructive interference, and double-metal defect layers to achieve MCC, integrating IR, laser, and visible light. These MCC films exhibit low emissivity in the dual IR bands (∼0.2 at 3–5 μm and ∼0.4 at 7.5–13 μm) for high-temperature thermal camouflage, low reflectance at 10.6 μm (∼0.3) for reducing laser signal, and demonstrate excellent insensitivity to angles and polarization. By varying the thickness of the resonant cavity, a wide color gamut in the visible light range is achieved, maintaining efficient IR and laser compatibility while integrating diverse structural colors. This work offers a promising and pattern-free method for MCC design, holding great potential in thermal management and camouflage.

## 1 Introduction

Nowadays, detection and guidance technologies are increasingly diversified, with advanced reconnaissance systems operating across multiple bands such as laser, IR, and visible light being integrated into joint applications [1], [2], [3], [4], [5], [6]. On the one hand, passive IR detection and active laser detection have collectively evolved into multimodal composite detection methods, extensively utilized in overcoming anti-camouflage challenges [7], [8], [9]. These methods are capable of simultaneously capturing the thermal radiation and echo signals from objects of interest [10], [11]. On the other hand, the resolution of reconnaissance satellites has reached up to 0.1 m, enabling the easy discrimination of color mismatches against the background [12]. Therefore, the development of a MCC device that encompasses visible light, IR, and laser has gained significant importance today [13], [14], [15].

However, traditional materials used for camouflage are barely capable of covering such a broad spectrum [16], [17], [18], [19]. This is due to conflicts in spectral requirements across visible, IR, and laser bands. Specifically, IR detectors primarily operate in the mid-wavelength band of 3–5 μm and the long-wavelength band of 7.5–13 μm, which correspond to the two atmospheric windows for IR radiation [20], [21]. Typically, there is an expectation for low IR emissivity to achieve efficient thermal radiation suppression within these bands for camouflage. However, laser detectors, which are active detection systems operating in the IR band, can receive return signals due to the high reflectance resulting from low emissivity. This necessitates minimizing laser reflectance to avoid signal return and detectable features. In the visible band, using multiple colors and matching with the background are the most effective methods for evading detection. The fundamental principle relies on regulating the reflectance peak within visible band to achieve a wide gamut, ensuring precise color integration with the background. However, it is challenging for integrating the customized visible spectra, high IR reflectance, and low laser reflectance in a single device [22].

Thus, there is a need for materials that can selectively manipulate electromagnetic waves across different spectra as desired. The angle-selective thermal emitter proposed by Li et al. [23] employs nanophotonic structures, which can selectively regulate electromagnetic waves ranging from the solar spectrum to the thermal infrared band, thereby providing new insights for radiative cooling and thermal camouflage. Most efforts focus on constructing hierarchical and periodic photonic structures to manipulate electromagnetic waves and achieve MCC. For example, Yang et al. [24] prepared a multilayer film structure using five materials for IR-laser-compatible camouflage. Ye et al. [25] fabricated a SiO_2_/Ge/SiO_2_/Ge film to obtain compatible IR thermal camouflage and management. Li et al. [26], [27], [28] proposed a series of photonic structures, achieving camouflage and thermal management that cover the near-infrared and full-band thermal infrared, which has greatly advanced the development of multi-band compatible camouflage technology. Moreover, metamaterials and metasurfaces represent an emerging strategy for achieving MCC [29], [30], [31], [32]. For instance, periodic arrays such as Al and Au disks [33], [34], [35], [36], apertures on dielectric/metal/dielectric films [37], Au cubes [38], and wavelength-scale grating structures [39], [40] are feasible ways to strike a balance in MCC. However, these approaches exhibit a weak ability to incorporate visible camouflage and are expensive, time-consuming, and challenging for large-scale fabrication in practical applications. Therefore, there is a high demand for pattern-free methods to achieve MCC that encompasses visible-IR-laser compatibility for practical applications.

Here, we propose two heterogeneous multilayer films with SiO_x_/Cu/SiO_2_/Pt (x = 1 or 2) structures for IR, visible, and laser MCC. Both designs can achieve multicolor, IR low emissivity, and single wavelength absorption in the IR band. The effective laser absorption at 10.6 μm with precise “spectral notch filtering” is realized by further optimizing the top layer with SiO. The various color appearances can be obtained by altering the resonant mode for structural color excitation without degrading IR low emissivity and high laser absorption. The MCC films possess several distinctive advantages: (1) wide gamut and low IR emissivity in the whole IR band (2.5–25 μm) for both photonic structures, and precise CO_2_ laser radar narrow absorption peak for SiO/Cu/SiO_2_/Pt multilayer film; (2) the pattern-free design enables the large-scale fabrication and high possible for implementing in practical camouflage; (3) the angle-/ polarization- independent characteristics ensuring the high-performance camouflage in all directions.

## 2 Results and discussion

### 2.1 MCC design principles and configuration

**Figure 1:**
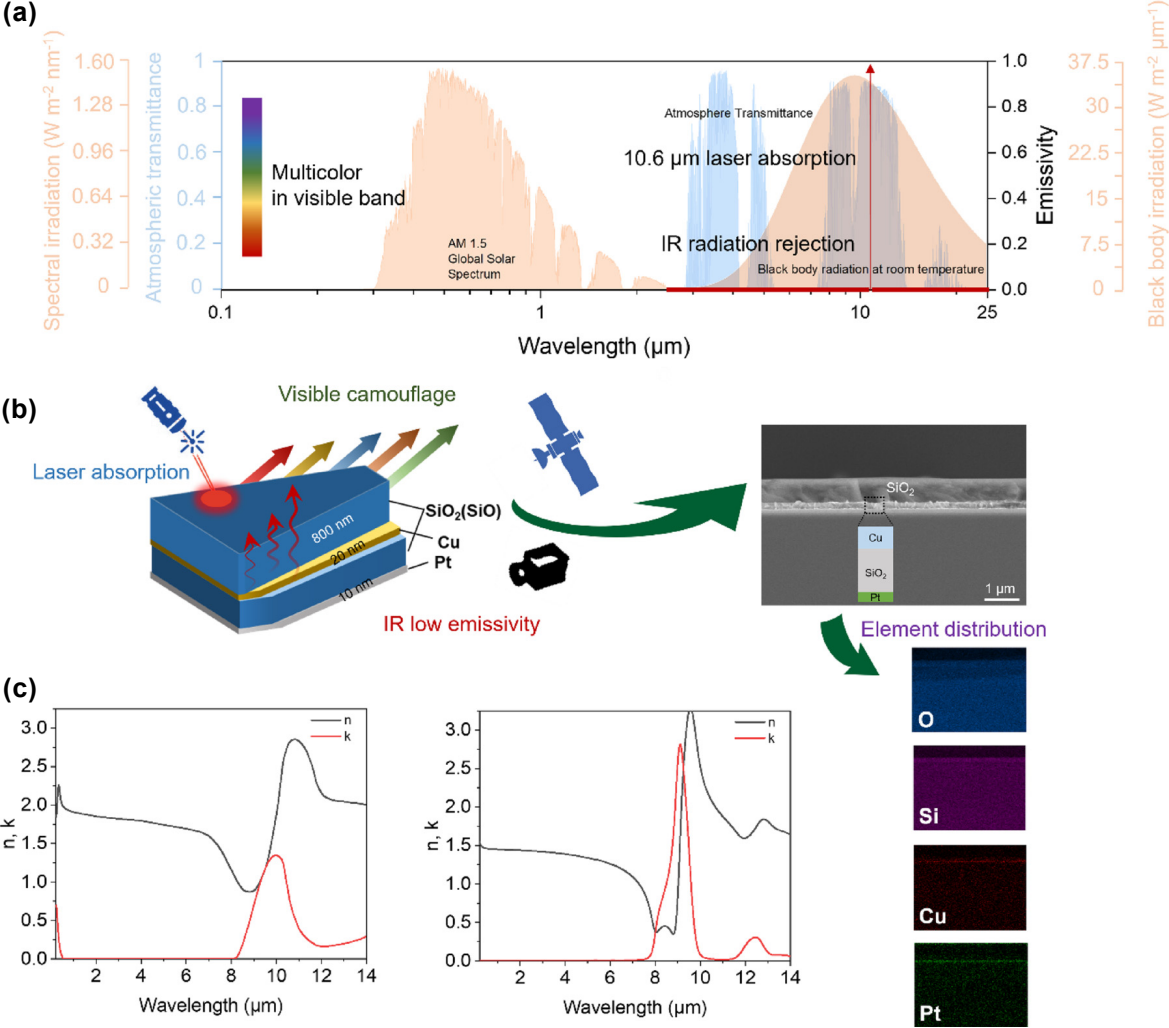
Spectra requirements of MCC for IR, visible, and laser and configuration. (a) The solar radiation intensity distribution, black body radiation intensity at 35 °C, atmospheric transmittance and ideal emissivity/absorptivity combined with multicolor appearance for MCC. The IR radiation rejection means low emissivity in the range from 2.5 to 25 μm. (b) Schematic of the designed MCC film (SiO_2_/Cu/SiO/Pt: 800 nm/20 nm/ 120 nm/ 10 nm), SEM image of the MCC film cross section, and the energy dispersive spectrometer mappings of the cross-section. (c) Refractive indexes of SiO (left) and SiO_2_ (right).

As shown in Figure 1a, the design of MCC films is contingent upon the spectral demands across various regimes, which are pivotal for masking the intrinsic characteristics of an object. Specifically, the MCC films must modulate the characteristic reflectance within the visible spectrum to broaden the color gamut, thereby facilitating the seamless integration of the target object with its surroundings. Additionally, these films should exhibit low IR emissivity within the blackbody radiation spectrum (2.5–25 μm), which is crucial for minimizing thermal emissions and evading IR detection. Furthermore, a narrow-band low-reflection gap should be engineered to counteract active laser detection at 10.6 μm. The challenge lies in the fact that a simple overlay of low IR emissivity and colorful coatings is insufficient due to the operation of single-wavelength laser detection within the IR spectrum. It will inevitably result in significant return signals. Therefore, a thorough consideration of selective spectral properties is imperative for achieving visible background integration, suppression of IR thermal radiation, and reduction of laser return signals.

The SiO_x_/Cu/SiO_2_/Pt (x = 1 or 2) multilayer heterogeneous film has been strategically engineered to modulate the tri-band spectrum, encompassing IR, visible, and laser-compatible camouflage capabilities (Figure 1b). The intricate coupling of these functional layers resolves spectral conflicts, enabling the achievement of multicolored appearances, broad-spectrum low IR emissivity, and precise low reflection at 10.6 μm. The top SiO_x_ layer serves as a laser absorption structure, offering ultra-wide IR transparency due to its narrow extinction index (*k*) peak within the IR band and a relatively low refractive index (*n*). Moreover, it behaves as a lossless dielectric within the visible band (Figure 1c) [41], [42]. These properties are essential prerequisites for achieving compatible camouflage with both IR and laser systems, as well as for seamless integration with multicolored appearances. The bottom Cu/SiO_2_/Pt cavity supports a variety of structural colors and maintains low IR emissivity. Here, the SiO_2_, acting as a spacer with varying thicknesses, can excite multiple resonant modes. The Pt layer functions as a reflective mirror, in conjunction with the Cu film, to ensure low IR emissivity and to form a resonant cavity.

As shown in Figure 1b, the cross-sectional scanning electron microscope (SEM) reveals a well-defined multilayer structure fabricated by electron-beam evaporation on quartz substrate. Notably, each layer of the film demonstrates a uniform morphology and excellent adhesion, with no evident signs of percolation.

### 2.2 Multicolor performance

**Figure 2:**
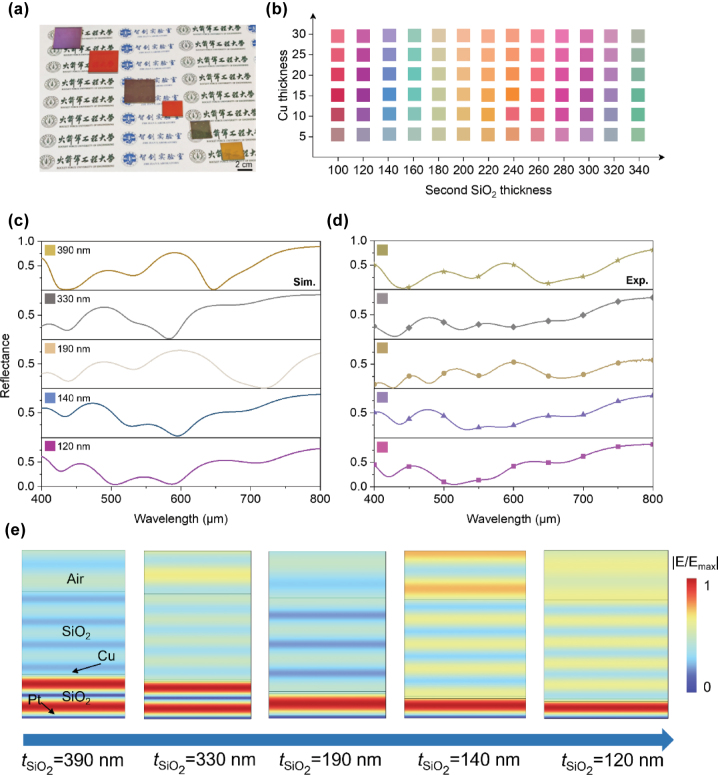
Multicolor and structural color mechanisms. (a) The photograph of the fabricated five different structural color samples. The thicknesses of the SiO_2_ spacers from the top-left corner to the bottom-right corner are 120 nm, 100 nm, 190 nm, 110 nm, 180 nm, and 210 nm, respectively. (b) The color palettes of the MCC film with different thickness of Cu and SiO_2_ spacer layer. (c–d) The calculated and measured visible reflection spectra of the designed MCC film with SiO_2_ spacer thickness of 120 nm, 140 nm, 190 nm, 330 nm, and 390 nm. Its corresponding color was also given. (e) Electric field distribution of the MCC film with different thickness of SiO_2_ spacer at the resonant absorption peak (620 nm, 570 nm, 730 nm, 600 nm, and 510 nm).

We successfully fabricate a series of multicolor MCC films by modulating the thickness of the SiO_2_ spacer layer, as shown in Figure 2a. Variations in the thickness of the intermediate SiO_2_ layer alter the optical path difference of light within this layer. Combined with the phase mutation caused by interfacial reflection, this leads to specific wavelengths of light satisfying the conditions for constructive interference. The dominant presence of such wavelengths in the reflected light results in the corresponding color. This approach allows us to achieve a spectrum of colors, including purple, wine red, brown, khaki, and orange, through the precise control of SiO_2_ spacer layer thickness. Furthermore, we demonstrate the scalability and color combination capabilities of our MCC films on a 4-inch silicon wafer, confirming their potential for large-scale applications. Utilizing a mask, we are able to create a vibrant, large-scale MCC film on a single substrate with varying SiO_2_ spacer thicknesses (Figure S1). To underscore the broad color gamut potential for full-color presentation, we calculate the structural color corresponding to SiO_2_ spacer thicknesses ranging from 100 nm to 340 nm (Figure 2b). When the thickness of Cu is set as 10 nm, the color spectrum can extend from red to green, covering nearly the entire visible range. Our studies also reveal that the Cu layer thickness is a critical parameter influencing the resulting structural color. However, variations in Cu thickness primarily affect the color’s brightness and saturation, with a limited capacity to significantly broaden the color palette. Notably, ultra-thin Cu films of less than 5 nm struggle to produce pure, bright, and saturated colors, whereas thicknesses exceeding 10 nm can effectively generate vivid structural colors (we select 20 nm for all samples fabrication and the color space can be seen in Figure S2).

Figure 2c–d depict the simulated and measured reflection spectra of the MCC film with varying thickness of SiO_2_ spacer layer and their corresponding color. Obviously, the simulated results on the same structure quantitatively corroborate the measured spectra, with the structural colors displayed in the insets exhibiting high similarity. The color is determined by the reflectance valley, which arises from resonant absorption. To further elucidate the structural color mechanisms of our MCC, we calculate the electric field distribution at the valley wavelength for these five structures. As illustrated in Figure 2e, the electric field resonance can be excited within the SiO_2_ spacer, leading to strong localized enhancement for effective electromagnetic wave absorption. Similar phenomena can also be observed in magnetic field distributions, where distinct magnetic resonance phenomena are evident within resonant cavities of varying thicknesses (Figure S3). It is precisely the co-resonance of electric and magnetic fields that triggers the absorption of electromagnetic waves. The double-metal layers of Cu and Pt attenuate the electromagnetic wave, confirming the role of the metal/dielectric/metal cavity in structural color generation (Figure S3). As the thickness of the SiO_2_ spacer increases, the resonant absorption and electromagnetic wave localization are enhanced.

We also calculate the structural color of SiO/Cu/SiO_2_/Pt structure, analyzing the trends by varying the thicknesses of the Cu and SiO_2_ spacer layers. The resulting color exhibits relatively low brightness, purity, and saturation, with a limited color palette (Figures S4 and 5). The observed differences stem from the increased opacity of SiO compared to SiO_2_, which limits the expansion of the color palette. Nonetheless, the SiO top layer remains effective for multicolor camouflage, enabling the creation of a diverse range of structural colors. Both structures, featuring SiO_2_ and SiO coatings, respectively, share the same mechanism for structural color generation, attributed to the resonant cavity formed by the Cu/SiO_2_/Pt configuration. Our design methodology can be readily adapted to various resonant cavities by substituting the bottom metal layer with alternative metals, thereby confirming its high efficiency and broad applicability for generating a wide spectrum of structural colors (Figure S6).

### 2.3 IR and laser camouflage

**Figure 3:**
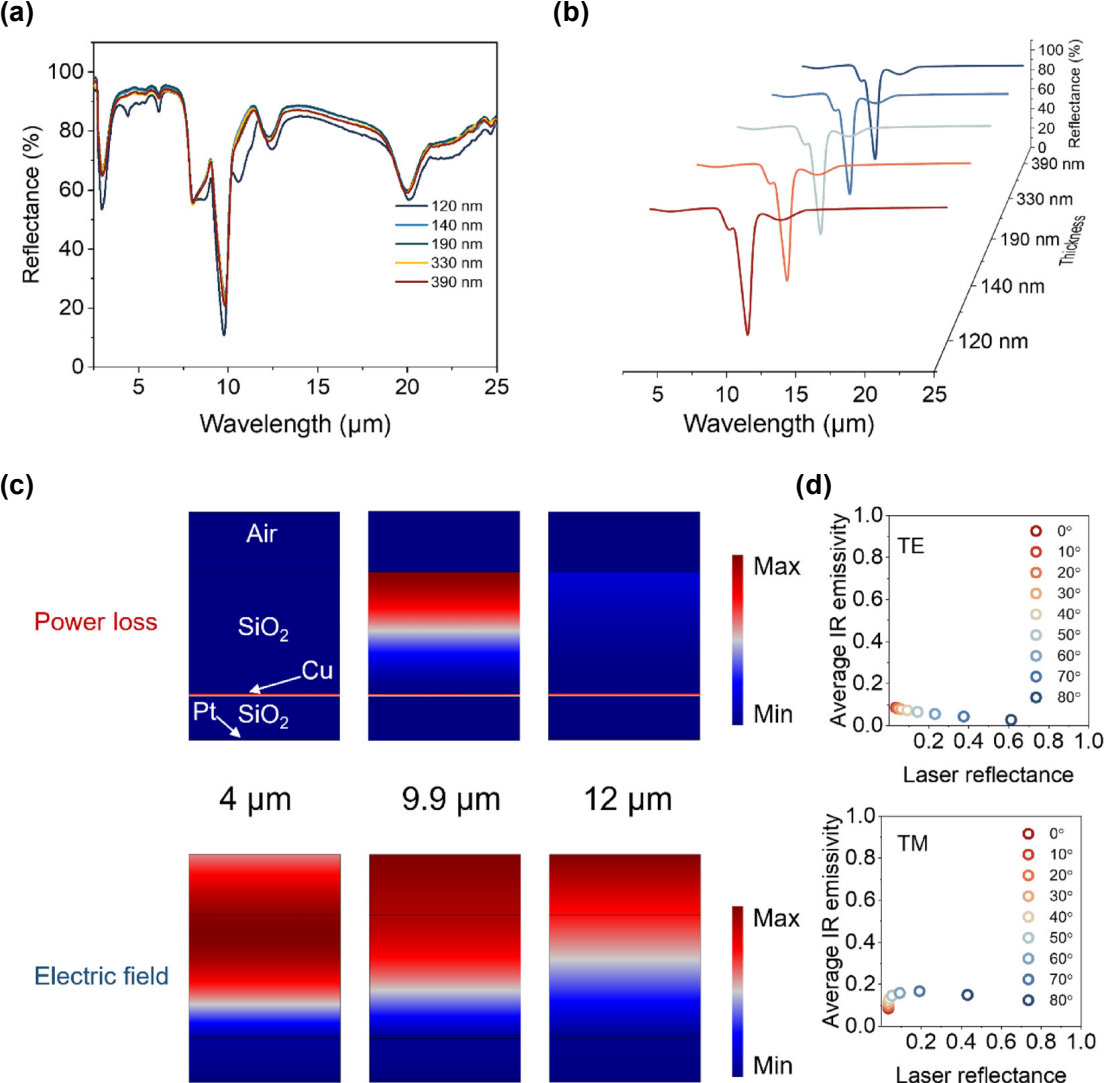
Ultra-wide low IR emissivity and compatible with laser camouflage for SiO_2_ top layer. (a–b) The measured (a) and simulated (b) IR spectra of MCC films with different thickness of SiO_2_ spacer layer (120 nm, 140 nm, 190 nm, 330 nm, and 390 nm). (c) The power loss and electric field distribution at 4 μm, 9.9 μm, and 12 μm. (d) The average IR emissivity and laser absorption at 9.9 μm under TE and TM modes with incident angles ranges from 0° to 80° (the thickness of SiO_2_ spacer is 270 nm).

The MCC films are capable of exhibiting a range of structural colors by adjusting the thickness of the resonant cavity, without compromising their low IR emissivity and high narrow-band laser absorption. We conduct measurements and simulations of the IR spectra for five different structural color films to assess their compatibility with multicolor applications (Figure 3a and b). As the cavity thickness increases, the IR spectra remain stable, demonstrating a thickness-independent characteristic. A significant decrease in reflectance is observed at 20 μm due to the strong phonon-polarization resonance of silica, where substrate effects are neglected in the simulations [43]. The measured and simulated results are in good agreement across the entire IR range. Our MCC films achieve an average IR emissivity of approximately 0.25 (2.5–25 μm) with a low laser reflectance of less than 0.2 at 9.9 μm, an emissivity of approximately 0.1 in the mid-wave IR (MWIR) region, and approximately 0.3 in the long-wave IR (LWIR) region (Figure S7).

The distribution of power loss and electric field are presented in Figure 3c, highlighting the laser absorption function of the SiO_2_ layer at 9.9 μm due to significant power loss. From the electromagnetic loss distribution at wavelengths of 4 μm and 12 μm, it can be observed that due to the absence of destructive interference effects, there is no power loss within the SiO_2_ film layer, which ensures the compatibility of low emissivity performance in the IR band. It is worth mentioning that electromagnetic waves are blocked by the Cu layer, and the electric field gradually weakens after entering the SiO_2_ film layer, thereby preventing IR light from entering the resonant cavity where structural colors are generated. This demonstrates the successful integration of IR, visible, and laser-compatible camouflage functions in our MCC film without interference. The power loss in the Cu layer at 4 μm and 9 μm is attributed to its relatively low *n* and high *k*, leading to minor partial absorption of IR [44]. However, this has a negligible impact on the IR low emissivity performance. To further investigate its performance in IR-laser compatibility, we find that the film maintains highly independent properties in both polarization and incident angles. As can be seen in Figure 3d, the MCC film exhibits ultra-wide angle-independence in both transverse electric (TE) wave and transverse magnetic (TM) wave up to 60°. The laser reflectance increases with the incident angle in both modes, leading to reduced performance in laser camouflage, while the IR low emissivity for thermal camouflage improves in TE mode and decreases in TM mode as the incident angle increases. However, the MCC film demonstrates more stable IR low emissivity compared to laser low reflectance as the incident angle increases. It can be explained that the low laser reflectance characteristic originates from the quarter-wavelength interference effect and exhibits strong angular sensitivity, whereas the low infrared emissivity arises from the synergistic effect of the dual metal layers and is independent of angle.

For practical laser camouflage applications, the 9.9 μm laser low reflectance characteristic is limited to specific laser detection systems that are not widely utilized. Therefore, it is necessary to shift the low reflectance valley to the operational wavelength of the CO_2_ laser, which is 10.6 μm. SiO exhibits a red-shift in the peaks of its *n* and *k* compared to SiO_2_, while maintaining similar optical parameters (Figure 1c). Consequently, replacing the SiO_2_ top layer with SiO can effectively shift the laser absorption peak to 10.6 μm, while preserving the IR low emissivity and multicolor compatibility of the film.

**Figure 4:**
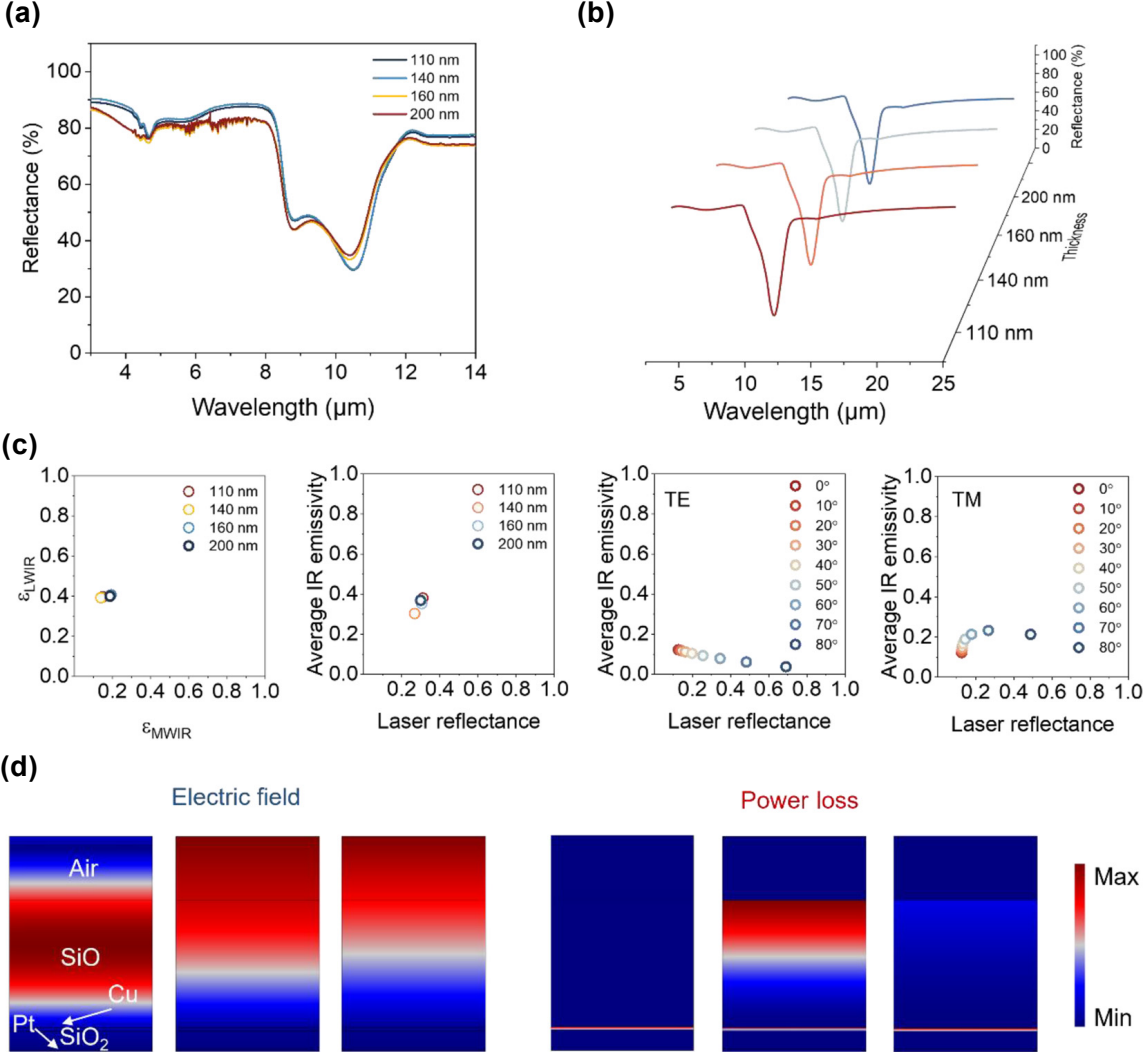
Ultra-wide low IR emissivity and compatible with laser camouflage for SiO top layer. (a–b) The measured (a) and simulated (b) IR spectra of MCC films with different thickness of SiO_2_ spacer layer (110 nm, 140 nm, 160 nm, and 200 nm). (c) The average IR emissivity in MWIR and LWIR, average mid-IR emissivity and laser absorption at 10.6 μm with different thickness of SiO_2_ spacer layer, and the IR-laser compatible performance under TE and TM modes with incident angles ranges from 0° to 80°. (d) The electric field and power loss distributions at 4 μm (left), 10.6 μm (middle), and 12 μm (right).

The multicolor capabilities of the SiO/Cu/SiO_2_/Pt structure are previously discussed. Here, we randomly selected four samples with varying thickness of the SiO_2_ spacer layer: purple (110 nm), cyan (160 nm), blue (140 nm), and pale yellow (200 nm). All the MCC films are fabricated with uniform cross-sectional morphology and clear boundaries (Figure S8). The IR and laser-compatible camouflage of four selected MCC films are experimentally demonstrated and simulated. Figure 4a and b show the measured and simulated emissivity spectra of the four selected samples in the IR band, the overall agreement of which implies the effectiveness of our methods. However, the laser low reflectance performance does not meet expectations due to oxidation of the SiO layer, which degrades the low reflectance at 10.6 μm. Additionally, an abrupt increase in emissivity at ∼9 μm is observed, originating from the phonon-polarization resonance of the quartz substrate. These factors contribute to a decrease in the IR and laser-compatible camouflage performance of the fabricated films. Fortunately, the resulting MCC films remain effective for achieving compatible camouflage. As illustrated in Figure 4c, we calculate the IR emissivity in MWIR (∼0.2) and LWIR (0.4) regions for these four samples, which can be utilized to suppress IR thermal radiation in two atmospheric windows. Moreover, all samples exhibit low laser reflectance at 10.6 μm (∼0.3) for preventing laser detection. For further verification, we evaluate their polarization (TE and TM) and incident angle independence. They exhibit similar independent properties to the SiO_2_ top layer structure, maintaining highly stable IR low emissivity under both polarization modes and up to 60° incident angles (Figure S9).

Their electric field and power loss distribution are presented in Figure 4d for elucidating compatible camouflage mechanisms. The electromagnetic wave can get into the SiO layer but be inhibited on Cu surface at 4 μm, 10.6 μm, and 12 μm. The SiO layer can effectively absorb electromagnetic wave at 10.6 μm due to strong power loss distribution. Specifically, since the thickness of metallic Cu (20 nm) is greater than the skin depth, it can effectively block IR thermal radiation across the entire IR band, so the electric field is basically concentrated in the film layers above the Cu layer (Figures S10 and 11). However, due to quarter-wavelength destructive interference, electromagnetic waves entering the SiO film layer can be effectively dissipated, achieving low reflectivity. Meanwhile, there is almost no power loss in other wavelength bands, thus ensuring that the Cu layer and Pt layer contribute to low IR emissivity.

### 2.4 Thermal camouflage

**Figure 5:**
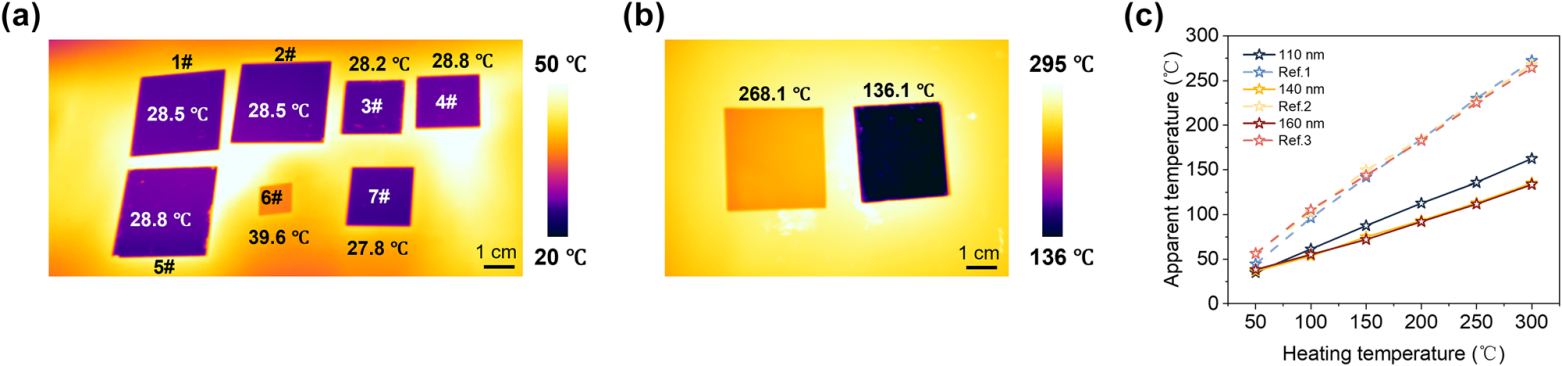
Thermal emittance suppression performance. (a) The IR images of samples with heating temperature of 50 °C (SiO_2_ spacer thickness: 1# and 2#, 110 nm; 3# and 4#, 140 nm; 5#, 160 nm; 6#, quartz; 7#, 200 nm). (b) The high temperature thermal camouflage performance with object temperature of 300 °C (left: quartz, right: MCC film with 110 nm SiO_2_ spacer). (c) The measured apparent temperature of the MCC film with different spacer thickness and the hot plate temperature as a function of the heating temperature. Quartz is chosen as reference sample for all measurements.

In order to evaluate the thermal radiation suppression ability for IR camouflage, thermal images are recorded for four SiO structures along with a quartz reference. All experimental samples exhibit approximate temperatures ranging from 27.8 to 28.8 °C, demonstrating excellent thermal camouflage performance compared to the quartz reference at 39.6 °C, resulting in an 11.8 °C decrease in radiative temperature (Figure 5a). We also verify their high-temperature camouflage performance when heated to 300 °C. The quartz temperature reaches 268.1 °C, while the MCC film maintains a low radiative temperature of 136.1 °C, indicating its suitability for high-temperature IR camouflage (Figure 5b). As shown in Figure 5c, as the heating temperature increases, the selected three MCC films exhibit similar low emissivity performance with a linear increase. Moreover, they can significantly suppress thermal radiation under different ambient temperatures and effectively reduce the intensity of thermal radiation from the target (Figure S12). The thermal camouflage performance becomes more pronounced with increasing temperature. All fabricated samples show consistent IR thermal camouflage performance, demonstrating the excellent compatibility of our design when considering IR, visible, and laser camouflage together.

## 3 Conclusions

In summary, we develop MCC films with visible, IR and laser compatible camouflage by thoroughly spectral design. First, we couple the heterostructures with wavelength difference to meet the spectral requirements in those three bands. The SiO_x_ laser absorber, structural color resonant cavity, and metal defect layer for IR thermal suppression function independently without interference. Second, various structural color MCC films exhibit almost equal compatible performance with polarization and angle independence. The mechanisms of structural color generation, laser absorption, and IR emissivity are elaborately analyzed. Third, we evaluate their performance in IR thermal suppression for high-temperature utilization. These MCC films with highly compatible camouflage for IR, visible, and laser pave the way for against advanced multimode detection.

## Supplementary Material

Supplementary Material Details
