# Supplementary material for: Structural-color-enabled multispectral heterostructure for infrared and laser camouflage
Source: Nanophotonics. 2025 Sep 4;14(19):3201–10. doi: 10.1515/nanoph-2025-0303 (PMC12455286; doi:10.1515/nanoph-2025-0303)
Supplement: Supplementary file 1 — Supplementary Material Details [file j_nanoph-2025-0303_suppl_001.docx]

**Supporting information for**

Structural-Color-Enabled Multispectral Heterostructure for Infrared and Laser Camouflage

*Wenhao Wang^1^, Long Wang^1^*, Tonghao Liu^1^, Yina Cui^2^, Liuying Wang^1^*, Gu Liu^1^, Yangming Pang^1^, Xu Wu^1^, Xinyu Zhu^1^, Xiaohui Chi^1^, Haoke Yang^1^, Xiaohu Wu^3^**

^1^Zhijian Laboratory, Rocket Force University of Engineering, Xi’an 710025, China

^2^Shaanxi Key Laboratory of Artificially-Structured Functional Materials and Devices, Air Force Engineering University, Xi’an 710051, China

^3^Thermal Science Research Center, Shandong Institute of Advanced Technology, Jinan 250100, China

Experimental section

Film fabrication

The deposition of SiO_2_, SiO, Cu and Pt layers was carried out with E-beam evaporation system (MEB-600, Beijing Chuangshiweina Technology Co., Ltd, China) on quartz and silicon wafer substrates. The deposition rates were 0.5 nm/s, 0.3 nm/s, 2 nm/s, and 1 nm/s, respectively. Before deposition, the substrate is ultrasonically cleaned with acetone, ethanol, and deionized water in sequence to remove contaminants. Then, evaporation is carried out after ensuring that the vacuum degree in the chamber is maintained above 1×10⁻4 Pa. During electron beam evaporation, a Hall ion source is used to introduce high-energy ion bombardment onto the substrate surface for auxiliary deposition.

Optical and thermal characterization

The optical reflectance spectra of the proposed MCC film with various thickness were measured by a spectrophotometer (UH4150, Hitachi). The IR reflectance spectra of the samples were measured with a Fourier transform IR (FTIR) spectrometer (Nicolet Fisher IS50). The IR images were recorded using an IR camera (FLIR, T560).

Simulation methods

The reflectance, electric field distribution, magnetic field distribution, and power loss density are simulated with a radio frequency module in COMSOL Multiphysics. The refractive indices of all materials were sourced from the built-in material library of COMSOL Multiphysics.

SEM observation

The SEM images and energy dispersive x-ray spectroscopy were performed by a field-emission scanning electron microscope (Carl Zeiss, Gemini360).

Color calculation

The XYZ tristimulus values are derived from the reflectance spectrum *R*(*λ*), illuminant spectral power distribution *S*(*λ*), and the CIE 1931 standard observer color-matching functions $\bar{x}$(*λ*), $\bar{y}$(*λ*), $\bar{z}$(*λ*). The calculation process can be seen as follows:

$X=k\cdot\sum_{\lambda} R(\lambda)\cdot S(\lambda)\cdot\bar{x}(\lambda)\cdot\Delta\lambda$ (1)

$Y=k\cdot\sum_{\lambda} R(\lambda)\cdot S(\lambda)\cdot\bar{y}(\lambda)\cdot\Delta\lambda$ (2)

$Z=k\cdot\sum_{\lambda} R(\lambda)\cdot S(\lambda)\cdot\bar{z}(\lambda)\cdot\Delta\lambda$ (3)

where Δ*λ* is the wavelength interval; *k* is a normalization factor ensuring Y=100 for a perfectly reflecting surface:

$k=\frac{100}{\sum_{\lambda} S(\lambda)\cdot\bar{y}(\lambda)\cdot\Delta\lambda}$ (4)

Then, CIELAB is calculated using XYZ values and a reference white point (typically D50: *X*_n_=96.72, *Y*_n_=100.000, *Z*_n_=81.427). The conversion can be calculated as follows:

$x=\frac{X}{X_{n}}$, $y=\frac{Y}{Y_{n}}$, $z=\frac{Z}{Z_{n}}$ (5)

$f(t)=\left\{\begin{matrix} t^{1/3} & \text{ if }t>\varepsilon^{3} (\varepsilon=6/29) \\ \frac{t}{3\varepsilon^{2}}+\frac{4}{29} & \text{ otherwise } \end{matrix}\right.$ (6)

$L^{*}=116\cdot f(y)-16$ (7)

$a^{*}=500\cdot[f(x)-f(y)]$ (8)

$b^{*}=200\cdot[f(y)-f(z)]$ (9)


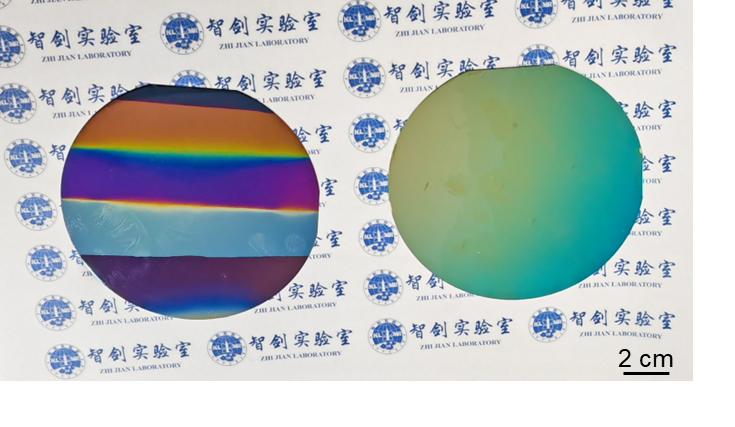


**Figure S1.** Photograph of the MCC film with various thickness of SiO_2_ spacer. The substrates are 4-in. Si wafer.

**Note:** For practical camouflage application scenarios, the ability to effectively achieve large-area fabrication is crucial. By altering the thickness of the resonant cavity and utilizing a mask, we have fabricated a mixture of colors including purple, blue, and orange on a single silicon wafer simultaneously.


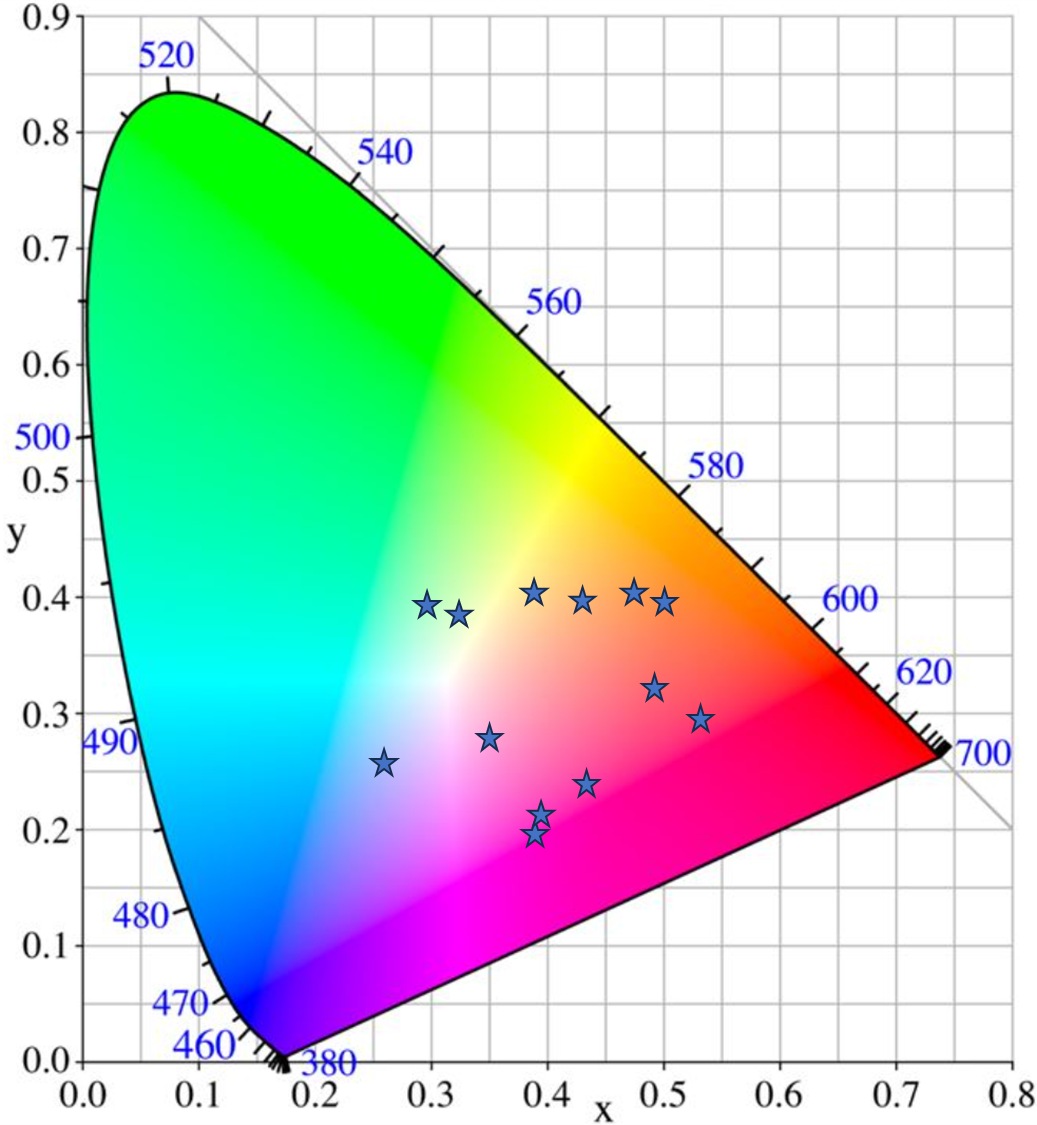


**Figure S2.** Simulated color of the MCC film (800 nm SiO_2_/ 20 nm Cu/ different thickness of SiO_2_ spacer/ 10 nm Pt) in CIE 1931 space.


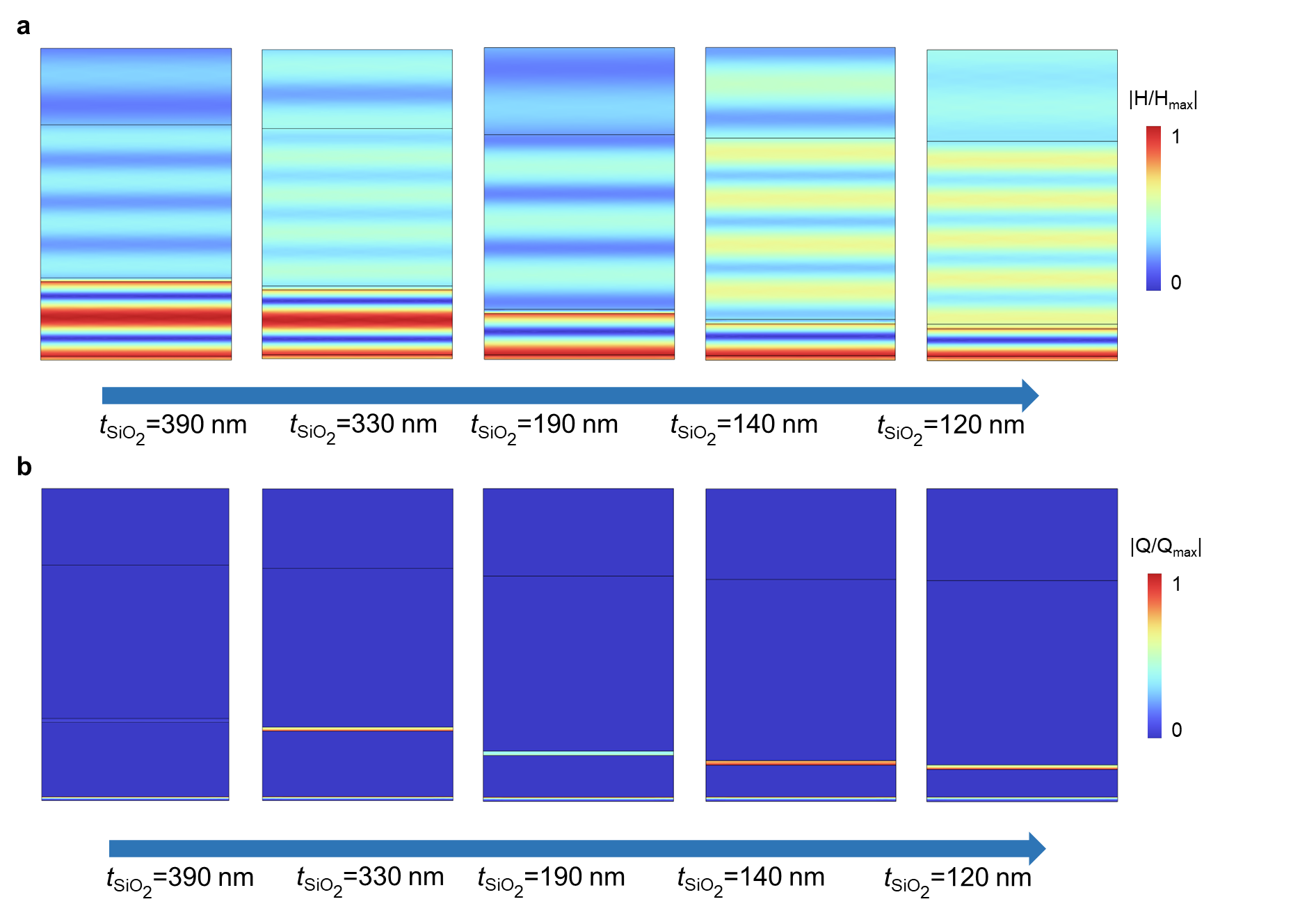


**Figure S3.** COMSOL Multiphysics analysis on electromagnetic wave behavior. (a) Magnetic field distributions for five different colors of MCC films with varying SiO_2_ spacer thicknesses. (b) Power loss distributions for five different colors of MCC films with varying SiO_2_ spacer thicknesses.


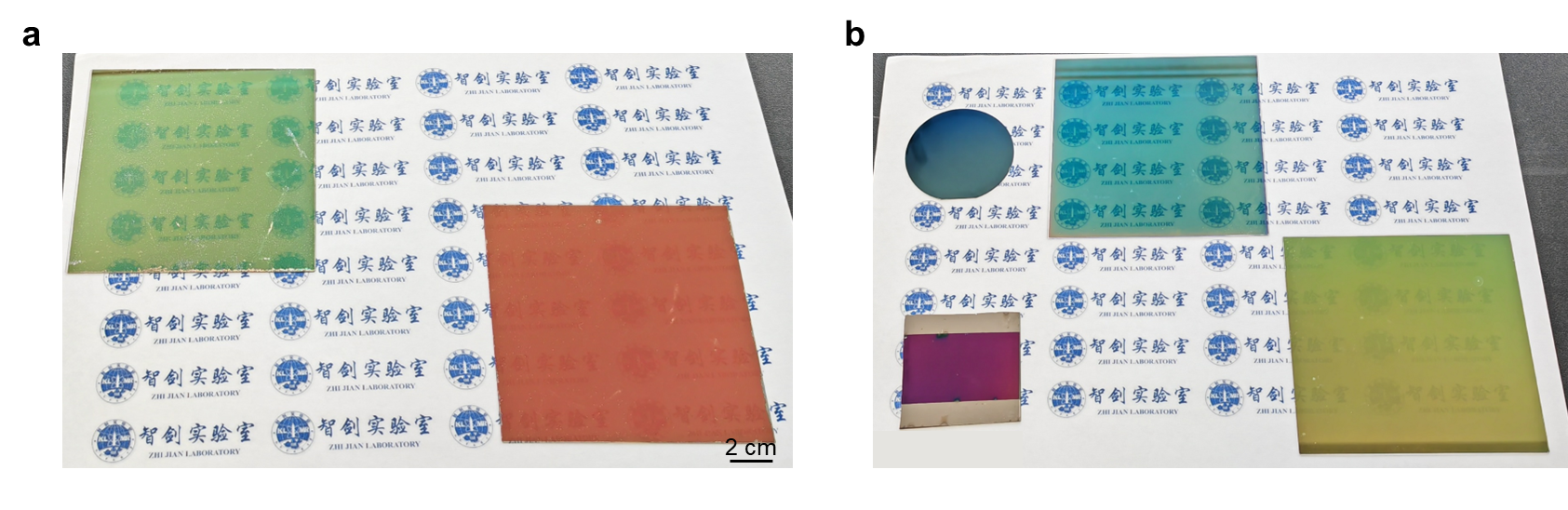


**Figure S4.** Photograph of the multicolor performance of SiO/Cu/SiO_2_/Pt structure. The substrates are 2-in. Si wafer and quartz.


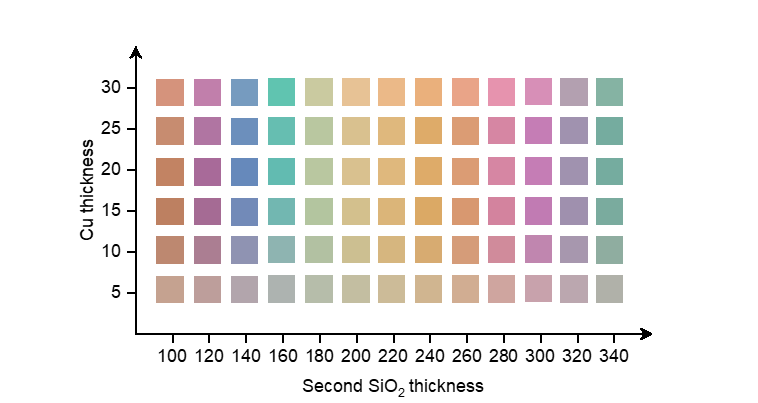


**Figure S5.** Color palette of SiO/Cu/SiO_2_/Pt structure with different thickness of SiO_2_ spacer and Cu film.

**Note:** To meet the practical requirements for stealth applications in laser detection, it is necessary to shift the low-reflection frequency from 9.9 μm to 10.6 μm. This requires an increase in the real part of the refractive index and a red-shift in the peak position of the imaginary part of the refractive index for the top dielectric layer. Consequently, we have designed a configuration based on SiO/Cu/SiO_2_/Pt, which effectively achieves precise low reflection at a wavelength of 10.6 μm. To evaluate the structural color performance, we have fabricated a series of thin films with various colors, as shown in Figure S4-5.


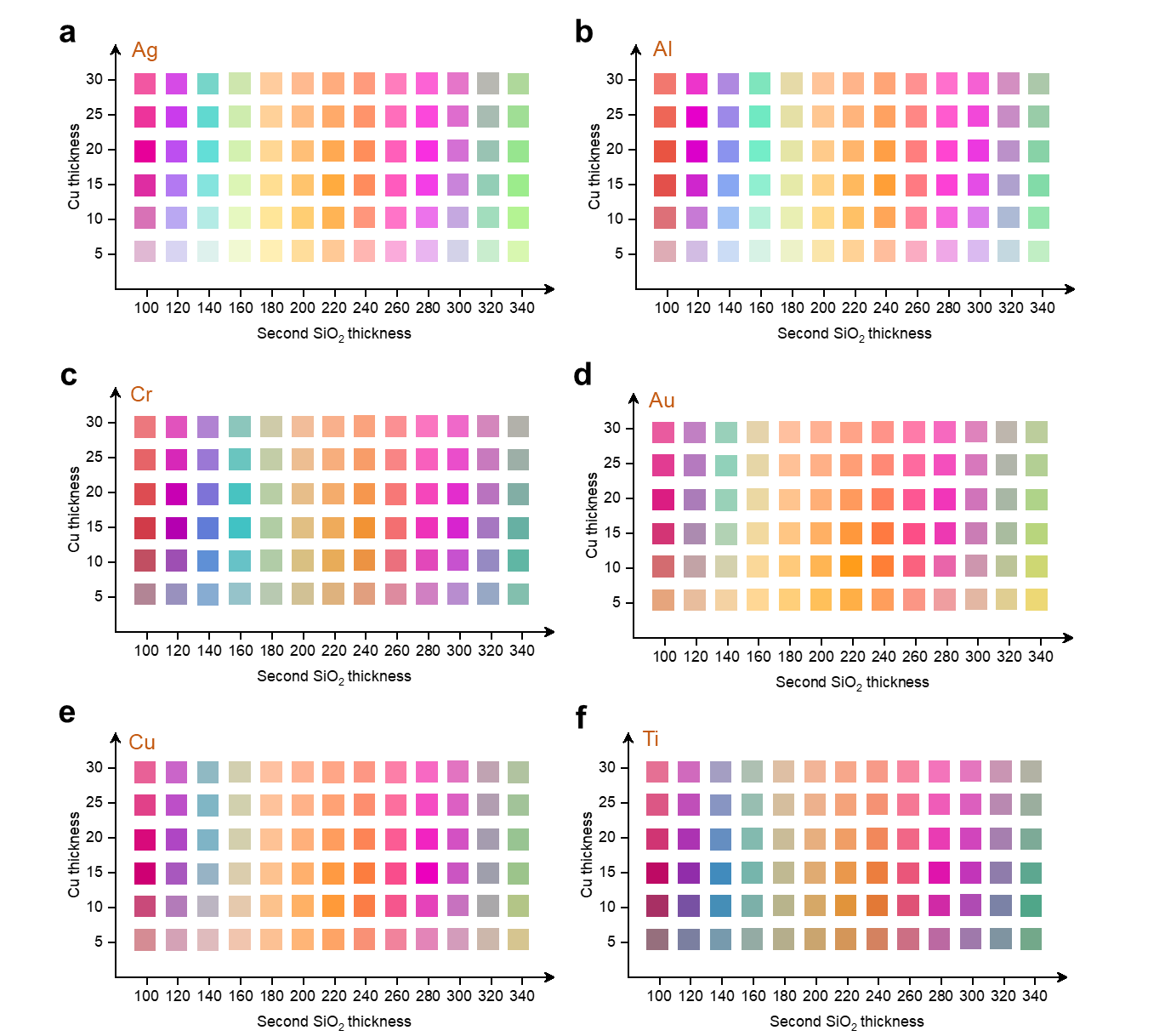


**Figure S6.** Structural color palette with different bottom metals. (a) Ag; (b)Al; (c) Cr; (d) Au; (e) Cu; (f) Ti.


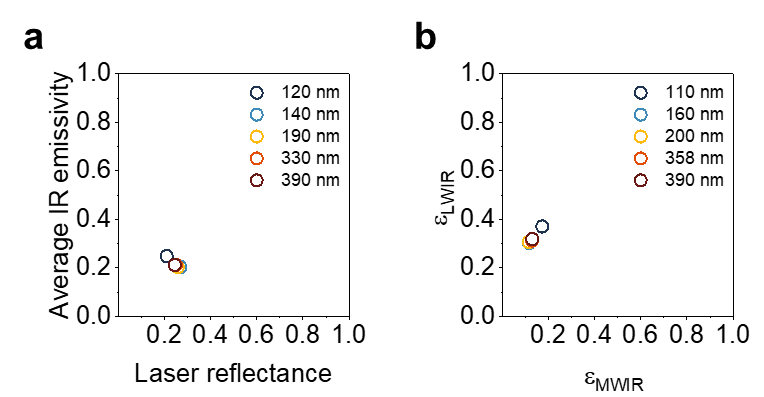


**Figure S7.** The laser and IR camouflage performance (a) and emissivity (b) in mid-wave IR (MWIR) and long-wave IR (LWIR) for five samples with varying thickness of SiO_2_ spacer (120 nm, 140 nm, 190 nm, 330 nm, 390 nm).

**Note:** To verify the consistent and efficient multispectral compatibility of different structural color films, we analyzed the IR low-emissivity (including performance in MWIR and LWIR) and laser low-reflection properties of films with various colors.


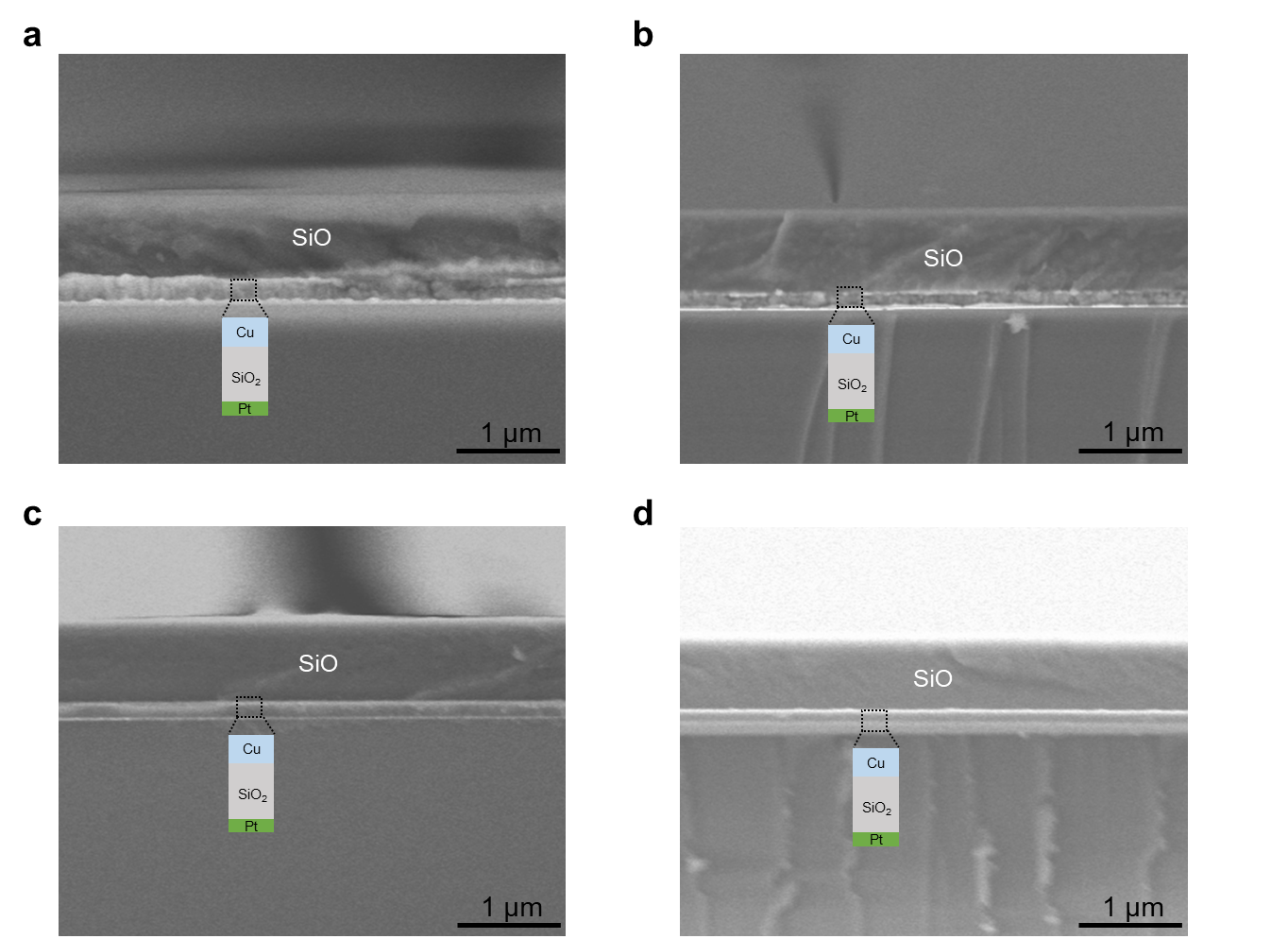


**Figure S8.** SEM images of the MCC films’ cross section (SiO/Cu/SiO_2_/Pt) with different thickness of SiO_2_ spacer. (a) 200 nm; (b) 160 nm; (c) 110 nm; (d) 140 nm.


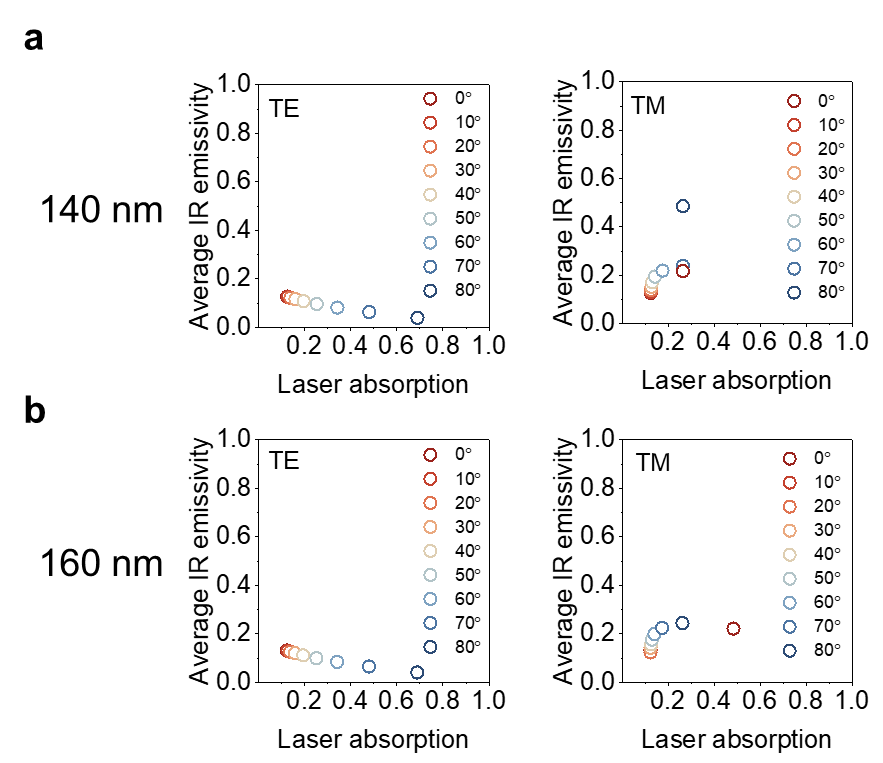


**Figure S9.** The laser and IR camouflage performance under TE and TM modes. (a) SiO/Cu/SiO_2_/Pt with SiO_2_ thickness of 140 nm; (b) SiO/Cu/SiO_2_/Pt with SiO_2_ thickness of 160 nm.


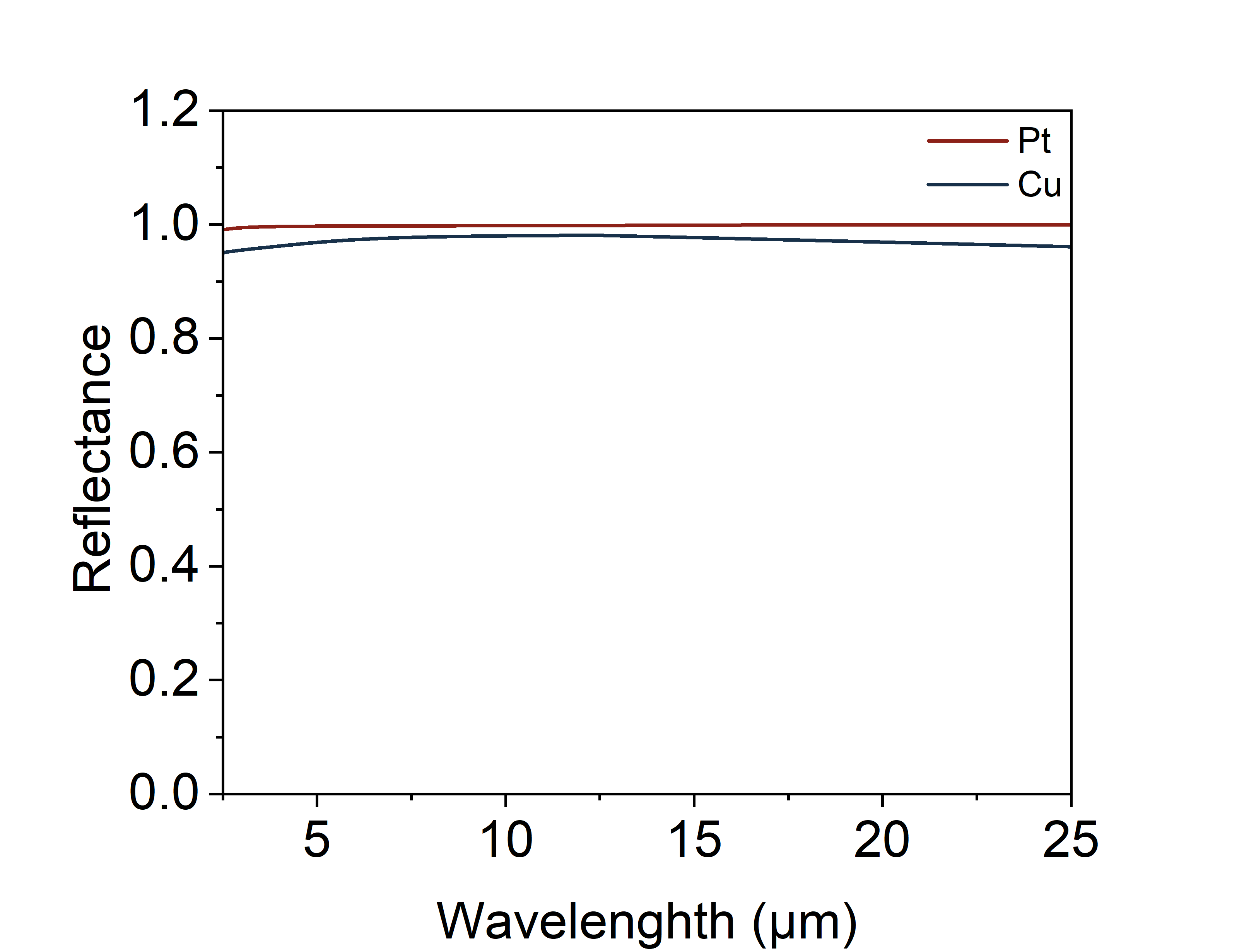


**Figure S10.** Simulated IR reflectance of single-layer Pt (10 nm) and Cu (20 nm) films.


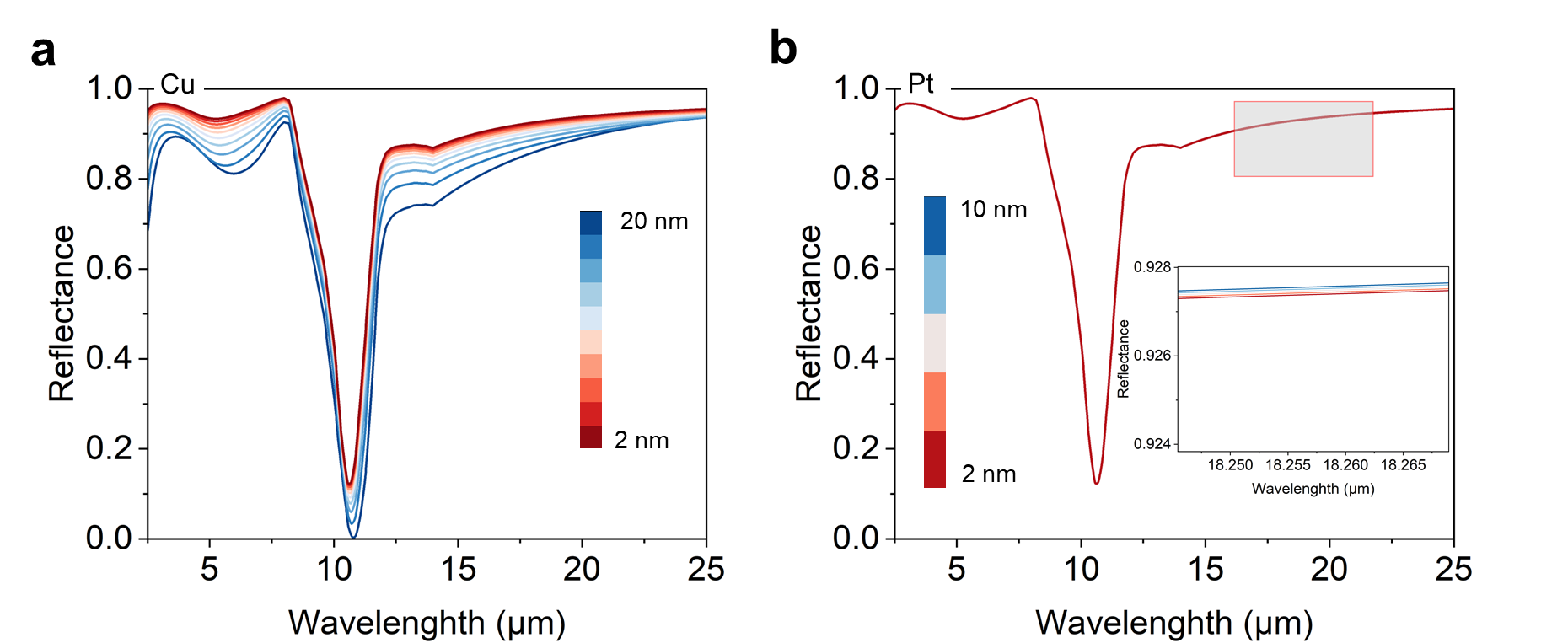


**Figure S11.** The IR reflectance of SiO/Cu/SiO_2_/Pt with varying thickness of (a) Cu and (b) Pt film. The Pt layer mainly plays a major role in generating structural colors, while the Cu layer dominates in contributing to low IR emissivity.


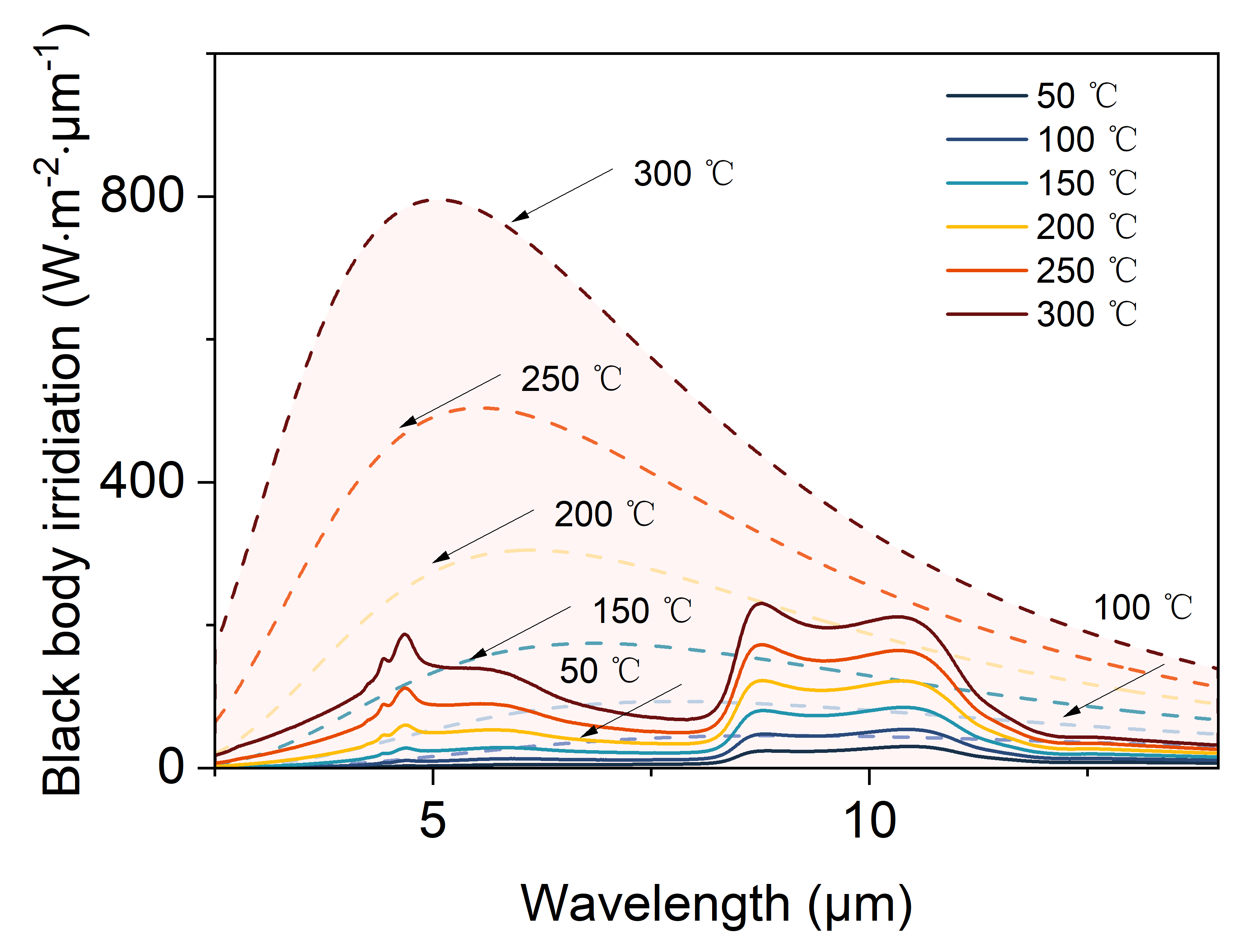


**Figure S12.** Curves of blackbody thermal radiation intensity and thermal radiation suppression performance at different temperatures (MCC film with SiO top layer and 110 nm SiO_2_ spacer). The dashed lines represent the blackbody thermal radiation intensity at different temperatures, while the solid lines indicate the thermal radiation suppression performance at different temperatures.
